# Estimating the Prevalence of a True Oligometastatic Disease

**DOI:** 10.3390/cancers15010214

**Published:** 2022-12-30

**Authors:** Petr Szturz, Pierluigi Bonomo, Jan B. Vermorken

**Affiliations:** 1 Medical Oncology, Department of Oncology, Lausanne University Hospital (CHUV), 1011 Lausanne, Switzerland; 2 Faculty of Biology and Medicine, University of Lausanne (UNIL), 1011 Lausanne, Switzerland; 3 Department of Radiation Oncology, Azienda Ospedaliero-Universitaria Careggi, 50134 Florence, Italy; 4 Department of Medical Oncology, Antwerp University Hospital, 2650 Edegem, Belgium; 5 Faculty of Medicine and Health Sciences, University of Antwerp, 2610 Antwerp, Belgium

To delineate a patient group with few distant metastases that could possibly benefit from a curative therapeutic strategy employing a local approach, the term oligometastatic disease (OMD) was introduced into the clinical practice almost 30 years ago [1]. Recently, Christ and coworkers characterized the imaging-based prevalence of OMD through a cross-sectional assessment at a large tertiary centre. The authors analysed state-of-the-art imaging modalities (7000 positron emission tomography scans and 130 complementary cranial magnetic resonance imaging examinations) performed at their institution between January and December 2020. Using a generally accepted criterion of a maximum of five distant lesions, they were able to calculate the prevalence of OMD, reaching up to 52% among 1155 patients with different types of disseminated cancer [2]. In addition, only one or two distant lesions were detected in 72% of the OMD cases. However, accumulating evidence from clinical trials and routine practice has shown that not all patients with OMD can be cured by eradicating all visible lesions. Moreover, both invasive and non-invasive local procedures carry a risk of severe toxicity and can delay systemic treatment, which, in some cases, may be more appropriate [3].

On the other hand, it can be hypothesized that a true OMD does not harbour any unrecognised microscopic dissemination, and thus, can be cured by local ablation alone. However, due to intrinsic difficulties in identifying such patients, no information on its prevalence is available. In this respect, the work by Christ et al. adds to the limited body of evidence, providing hypothesis-generating data about OMD prevalence based on modern imaging methods and periodic assessments [2]. Further building on these results, we performed a literature search to estimate the prevalence of a true OMD by identifying large-scale studies involving patients undergoing surgical resection of a limited number of distant metastases with a curative intent (Table 1). We selected only studies reporting 5- and 10-year survival rates to comply with the condition of a potential cure conveyed by local treatment. Probably, the most accurate estimates can be obtained from long-term disease-free survival (DFS) outcomes. These data are available for patients with disseminated colorectal carcinoma treated with hepatic metastasectomy and show almost a plateau between 5 and 10 years with a DFS of about 25% and 20%, respectively [4,5]. These milestone rates roughly correspond to an overall survival (OS) of around 40% and 25%, making the OS numerically higher than the DFS, as it also involves patients living with active cancer [4]. Interestingly, we found such OS results in the majority of other available datasets of patients with epithelial tumour types and sarcomas undergoing pulmonary metastasectomy [6,7,8]. In addition, 5- and 8-year OS estimates of 42% and 27%, respectively, were also yielded in the randomized SABR-COMET trial that explored the addition of stereotactic body radiotherapy to the standard-of-care therapy across different tumour types, mostly of epithelial origin. Another notable finding in this study was the estimated rate of long-term progression-free survival reaching a plateau at 21% between 4 and 8 years from randomization [9].

Altogether, these findings suggest that a long-term DFS of about 20% after local ablation of OMD may be relevant for the majority of epithelial tumour types and sarcomas. At the same time, we would like to point out that prognoses might be different in some less frequent cancer types involving malignant melanomas and germ-cell tumours, and that the number of metastases represents a relevant stratification factor with the best survival outcomes seen in patients with a single distant lesion, as summarized in Table 1.

In conclusion, about one fifth of OMD cases, corresponding to the assumed cure rate of 20% (long-term DFS), may present with a true OMD which, after combining with the results from Christ et al. (52% of OMD among all metastatic cases), corresponds to a prevalence of a true OMD of about 10% among all metastatic cancer cases. In these patients, local therapy alone may be the optimal approach to treat distant metastases. In this respect, we would like to underline that a distinction between an OMD and a true OMD pertains particularly to de novo (synchronous) oligometastases and oligorecurrent (metachronous) metastases, but is less valid for use in some specific clinical scenarios that probably have a different biology and include oligoprogression, where few distant lesions progress in an otherwise controlled polymetastatic setting, and oligopersistence, where few distant lesions persist after an otherwise successful treatment of a polymetastatic disease, typically with systemic treatment. Therefore, the use of a common language in the classification of OMD should be warranted in all future investigations as well as in all efforts aimed at characterizing this limited state of dissemination from a translational perspective [10,11].

## Figures and Tables

**Table 1:** Long-term survival outcomes in patients with oligometastatic disease enrolled in large-scale studies or meta-analyses.

First Author, Year, Reference	Data Source	Survival Analysis								
		Total Population (TP)/Subgroup (SG)	Tumour Type	Percentage of Complete Resections	Percentage of Oligometastatic Cases (Number of Metastases)	Site of Metastases (Treatment)	5-Year OS	10-Year OS	5-Year DFS	10-Year DFS
Kanas, 2012 [4]	meta-analysis	TP: 20,745 pts (116 stds)	colorectal cancer	-	-	-	-	-	-	-
		SG: 86 stds		-	-	various (HM)	38% *	-	-	-
		SG: 15 stds		-	-	liver only (HM)	38% *	-	-	-
		SG: 21 stds		-	100% (1)	liver only (HM)	45% *	-	-	-
		SG: 20 stds		-	-	−(HM)	-	26% *	-	-
		SG: 26 stds		-	-	−(HM)	-	-	25% *	-
		SG: 6 stds		-	-	−(HM)	-	-	-	20% *
Creasy, 2018 [5]	MSKCC	TP: 1211 pts	colorectal cancer	91%	88% (≤3)	93% liver only (HM)	-	-	-	16%
		SG: 588 pts		-	100% (1)	−(HM)	-	-	-	23% †
		SG: 477 pts		-	100% (2–4)	−(HM)	-	-	-	21% †
Pastorino, 1997 [6]	IRLM	TP: 5206 pts	epithelial (43%), sarcoma (42%), germ-cell (7%), melanoma (6%), other (2%)	88%	72% (≤3)	lung only (PM) ‡	-	-	-	-
		SG: 4572 pts	-	100%	74% (≤3)		36%	26%	-	-
		SG: 2169 pts	-	100%	100% (1)		43%	31%	-	-
		SG: 1226 pts	-	100%	100% (2–3)		34%	24%	-	-
		SG: 1984 pts	epithelial	100%	-		37%	21%	-	-
		SG: 1917 pts	sarcoma	100%	-		31%	26%	-	-
		SG: 318 pts	germ-cell	100%	-		68%	63%	-	-
		SG: 282 pts	melanoma	100%	-		21%	14%	-	-
Friedel, 2002 [7]	IRLM	TP: 467 pts	breast cancer	84%	87% (≤3)	lung only (PM) ‡	38%	22%	-	-
		SG: 272 pts		100%	100% (1)		44%	23%	-	-
Casi-raghi, 2011 [8]	IRLM	TP: 575 pts	epithelial (76%), sarcoma (16%), germ-cell (6%), melanoma (2%)	85%	71% (≤3)	lung only (PM)	43%	27%	-	-
		SG: 490 pts	-	100%	-		46%	29%	-	-

Abbreviations: MSKCC—prospectively maintained database at Memorial Sloan Kettering Cancer Center; IRLM—retrospective analysis of the International Registry of Lung Metastases (single-centre study by Casiraghi et al.); stds—studies; pts—patients; HM—hepatic metastasectomy; PM—pulmonary metastasectomy; OS—overall survival; DFS—disease-free survival; * median; † all patients survived for at least 10 years, but a minority of them (not specified by the authors but less than 25%) might have a shorter DFS (at least 3 years); ‡ some patients could have had curative-intent treatment of the primary tumour and extrapulmonary metastases (if there were any) before or simultaneously with pulmonary metastasectomy.
